# Vaccination Coverage with Selected Vaccines and Exemption Rates Among Children in Kindergarten — United States, 2021–22 School Year

**DOI:** 10.15585/mmwr.mm7202a2

**Published:** 2023-01-13

**Authors:** Ranee Seither, Kayla Calhoun, Oyindamola Bidemi Yusuf, Devon Dramann, Agnes Mugerwa-Kasujja, Cynthia L. Knighton, Carla L. Black

**Affiliations:** ^1^Immunization Services Division, National Center for Immunization and Respiratory Diseases, CDC; ^2^Certified Technical Experts, Inc., Montgomery, Alabama; ^3^Association of Schools and Programs of Public Health, Washington, DC.

State and local school vaccination requirements protect students and communities against vaccine-preventable diseases (*1*). This report summarizes data collected by state and local immunization programs* on vaccination coverage and exemptions to vaccination among children in kindergarten in 49 states^†^ and the District of Columbia and provisional enrollment or grace period status for kindergartners in 27 states^§^ for the 2021–22 school year. Nationwide, vaccination coverage with 2 doses of measles, mumps and rubella vaccine (MMR) was 93.5%^¶^; with the state-required number of diphtheria, tetanus, and acellular pertussis vaccine (DTaP) doses was 93.1%**; with poliovirus vaccine (polio) was 93.5%^††^; and with the state-required number of varicella vaccine doses was 92.8%.^§§^ Compared with the 2020–21 school year, vaccination coverage decreased 0.4–0.9 percentage points for all vaccines. Although 2.6% of kindergartners had an exemption for at least one vaccine,^¶¶^ an additional 3.9% who did not have an exemption were not up to date with MMR. Although there has been a nearly complete return to in-person learning after COVID-19 pandemic-associated disruptions, immunization programs continued to report COVID-19–related impacts on vaccination assessment and coverage. Follow-up with undervaccinated students and catch-up campaigns remain important for increasing vaccination coverage to prepandemic levels to protect children and communities from vaccine-preventable diseases.

As mandated by state and local school entry requirements, parents provide children’s vaccination or exemption documentation to schools, or schools obtain records from state immunization information systems. Federally funded immunization programs work with departments of education, school nurses, and other school personnel to assess vaccination and exemption status of children enrolled in public and private kindergartens and to report unweighted counts, aggregated by school type, to CDC via a web-based questionnaire in the Secure Access Management system, a federal, web-based system that provides authorized personnel with secure access to public health applications operated by CDC. CDC uses these counts to produce state- and national-level estimates of vaccination coverage among children in kindergarten. During the 2021–22 school year, 49 states and the District of Columbia reported coverage with all state-required vaccines and exemption data for public school kindergartners; 48 states and the District of Columbia reported coverage with all state-required vaccines and exemption data for private school kindergartners.*** Data from cities were included with their state data. State-level coverage and national and median coverage with the state-required number of DTaP, MMR, polio, and varicella vaccine doses are reported. Hepatitis B vaccination coverage is not included in this report but is available at SchoolVaxView (*2*). Twenty-seven states reported the number of kindergartners who were attending school under a grace period (attendance without proof of complete vaccination or exemption during a set interval) or provisional enrollment (school attendance while completing a catch-up vaccination schedule). All counts were current as of the time of the assessment.^†††^ National estimates, medians, and summary measures include only U.S. states and the District of Columbia.

Vaccination coverage and exemption estimates were adjusted on the basis of survey type and response rate.^§§§^ National estimates measure coverage and exemptions among all kindergartners, whereas medians indicate the midpoint of state-level coverage, irrespective of population size. During the 2021–22 school year, immunization programs reported 3,837,259 children enrolled in kindergarten in 49 states and the District of Columbia.^¶¶¶^ Reported estimates are based on 3,543,080 (92.2%) children who were surveyed for vaccination coverage, 3,688,904 (96%) surveyed for exemptions, and 2,487,284 (65%) surveyed for grace period and provisional enrollment status. Potentially achievable coverage with MMR (the sum of the percentage of children who were up to date with 2 doses of MMR and those not up to date but with no documented vaccination exemption) was calculated for each state. Nonexempt students include those who were provisionally enrolled in kindergarten, in a grace period, or otherwise without documentation of complete vaccination. SAS software (version 9.4; SAS Institute) was used for all analyses. This activity was reviewed by CDC and was conducted consistent with applicable federal law and CDC policy.****

Vaccination assessments varied by state because of differences in required vaccines and required numbers of doses, vaccines assessed, methods of data collection, and data reported (Supplementary Table 1, https://stacks.cdc.gov/view/cdc/123203). Kindergartners were considered up to date with a given vaccine if they received all doses required for school entry, except in eight states^††††^ that reported kindergartners as up to date for any vaccine only if they had received all doses of all vaccines required for school entry. States were asked to report any COVID-19–related impact on kindergarten vaccination measurement and coverage through a combination of structured responses and open-ended questions.

Nationally, 2-dose MMR coverage was 93.5% (range = 78.0% [Alaska] to 98.0% [New York]), with coverage of ≥95% reported by 13 states and <90% by nine states and the District of Columbia (Table). DTaP coverage was 93.1% (range = 78.0% [Alaska] to 98.3% [Virginia]); coverage of ≥95% was reported by 14 states and of <90% by 12 states and the District of Columbia. Polio vaccination coverage was 93.5% (range = 77.1% [Alaska] to 97.6% [Louisiana and Nebraska]), with coverage of ≥95% reported by 13 states and <90% by 10 states and the District of Columbia. Varicella vaccination coverage nationally was 92.8% (range = 76.1% [Alaska] to 98.0% [West Virginia]), with 12 states reporting coverage ≥95% and nine states and the District of Columbia reporting <90% coverage. Coverage decreased in most states for all vaccines compared with the 2020–21 school year (Supplementary Figure, https://stacks.cdc.gov/view/cdc/123205).

**TABLE:** Estimated* coverage^†^ with measles, mumps, and rubella; diphtheria, tetanus, and acellular pertussis; poliovirus; and varicella vaccines; grace period or provisional enrollment^§^; and any exemption^¶^^,^** among kindergartners, by immunization program — United States,^††^ 2021–22 school year

Immunization program	Kindergarten population^§§^	Surveyed,^¶¶^ %	2 Doses MMR,*** %	5 Doses DTaP,^†††^ %	4 Doses polio,^§§§^ %	2 Doses VAR,^¶¶¶^ %	Grace period or provisional enrollment, %	Any exemption, %	Percentage point change in any exemption, 2020–2021
National estimate****	3,837,259	92.2	93.5	93.1	93.5	92.8	2.4	2.6	0.4
Median****	—	—	92.9	92.0	92.7	92.6	1.9	2.7	0.2
**U.S. jurisdictions**									
Alabama^††††,§§§§^	60,332	100.0	≥94.9	≥94.9	≥94.9	≥94.9	NP	1.7	0.4
Alaska^§§§§,¶¶¶¶^	9,790	76.2	78.0	78.0	77.1	76.1	NR	4.6	0.6
Arizona*****	83,463	97.0	90.6	90.5	90.9	94.6	NR	6.8	1.3
Arkansas^†††††^	39,358	96.3	92.5	91.3	91.4	91.9	7.5	2.5	0.5
California^§§§§,^*****^,†††††^	512,144	98.4	96.3	95.7	96.2	96.0	1.2	0.2	−0.3
Colorado	66,900	97.5	88.4	89.1	88.8	87.6	≥0.6	≥3.2	−1.0
Connecticut^††††,§§§§^	35,451	100.0	95.7	96.0	96.0	95.5	NP	2.3	−0.3
Delaware^§§§§,†††††^	11,181	9.5	96.4	96.4	97.1	95.7	NR	1.2	−1.2
District of Columbia^††††,§§§§^	8,959	100.0	82.0	82.2	84.7	81.0	NR	0.5	0.2
Florida^§§§§,§§§§§^	229,432	97.8	≥91.7	≥91.7	≥91.7	≥91.7	4.3	3.9	0.8
Georgia^††††,§§§§^	118,742	100.0	≥83.2	≥83.2	≥83.2	≥83.2	0.4	4.7	1.8
Hawaii^§§§§^	13,368	6.7	94.3	92.5	93.2	90.2	<0.1	3.4	0.6
Idaho	23,854	99.6	83.9	83.5	84.0	83.4	1.8	9.8	1.6
Illinois^††††,§§§§^	137,699	100.0	92.1	91.9	91.9	91.8	NR	≥1.7	NA
Indiana^§§§§,§§§§§^	83,198	75.1	92.1	84.0	89.3	91.7	NR	2.4	0.5
Iowa^††††,§§§§^	40,111	100.0	≥90.6	≥90.6	≥90.6	≥90.6	5.4	2.4	0.2
Kansas^§§§§,†††††,§§§§§,¶¶¶¶¶^	36,526	29.5	91.1	90.0	92.2	90.4	NR	2.3	0.3
Kentucky^§§§§,†††††,§§§§§^	59,233	91.5	≥86.5	≥87.1	≥87.8	≥85.6	NR	1.3	0.3
Louisiana^††††^	66,518	100.0	93.7	96.2	97.6	91.4	NP	1.1	0
Maine	12,881	91.6	96.7	96.3	96.5	95.5	NR	1.8	−2.7
Maryland^§§§§,†††††^	53,866	98.4	93.9	88.6	94.8	92.7	NR	1.5	0.6
Massachusetts^††††,§§§§,†††††^	65,582	100.0	96.2	96.1	96.0	95.7	NP	1.0	−0.1
Michigan^††††^	114,251	100.0	93.6	94.1	94.8	93.6	0.7	4.5	0.8
Minnesota	69,403	98.7	89.0	89.0	89.3	88.7	NR	≥3.7	0.9
Mississippi^††††,§§§§,^*****	38,653	100.0	≥93.5	≥93.5	≥93.5	≥93.5	1.3	0.1	0
Missouri^††††,§§§§^	71,034	100.0	91.6	91.5	91.9	91.2	NR	≥3.0	0.5
Montana	NR	NA	NR	NR	NR	NR	NR	NR	NA
Nebraska^§§§§,†††††^	25,018	99.5	96.2	96.6	97.6	95.5	1.9	2.5	0.3
Nevada^§§§§^	36,855	99.2	92.7	91.5	92.2	92.1	3.1	4.8	0.4
New Hampshire^††††,§§§§,§§§§§^	12,157	100.0	≥88.7	≥88.7	≥88.7	≥88.7	5.2	3.4	0.6
New Jersey^††††,§§§§,§§§§§^	104,240	100.0	≥94.1	≥94.1	≥94.1	≥94.1	1.3	2.6	0.4
New Mexico^††††,§§§§^	20,736	100.0	94.3	94.0	94.3	93.6	0.4	1.4	0.5
New York (including New York City)^§§§§,^*****	195,377	99.3	98.0	97.3	97.4	97.4	2.0	0.1	0
New York City^§§§§,^*****	82,938	99.8	97.3	96.5	96.4	96.7	1.7	0.1	0
North Carolina^§§§§,†††††,§§§§§^	118,191	78.5	96.1	96.0	96.1	95.9	1.1	1.9	0.4
North Dakota	10,755	96.6	91.5	91.4	91.7	91.2	NR	5.3	1.1
Ohio	139,077	91.9	88.3	88.5	88.9	87.9	7.4	3.0	0.5
Oklahoma^†††††^	54,042	84.3	90.9	91.1	91.9	95.5	NR	3.5	1.1
Oregon^††††,†††††^	41,538	100.0	93.0	92.0	92.3	94.7	NR	7.0	1.6
Pennsylvania	139,558	94.9	95.0	95.4	95.1	94.8	NR	3.3	0.6
Rhode Island^§§§§,†††††,§§§§§^	11,002	96.9	97.3	97.0	97.1	97.0	NR	1.2	0.2
South Carolina^§§§§,¶¶¶¶¶^	58,276	27.2	92.7	91.0	91.9	92.4	3.4	3.4	1.0
South Dakota^††††,§§§§^	12,251	100.0	93.7	93.2	93.6	91.9	NR	3.5	0.1
Tennessee^††††,§§§§,§§§§§^	79,120	100.0	95.8	95.2	95.4	95.4	1.9	2.4	0.5
Texas (including Houston)^†††††,§§§§§^	389,037	99.3	94.0	93.7	94.0	93.5	1.8	2.9	0.6
Houston^†††††,§§§§§^	40,123	99.3	88.2	88.3	88.3	87.8	1.2	1.5	0.2
Utah^††††^	48,995	100.0	90.0	89.6	90.0	92.8	2.0	7.4	2.3
Vermont^††††,§§§§^	6,126	100.0	93.4	92.9	93.1	92.6	6.8	3.3	0.1
Virginia^§§§§,¶¶¶¶¶^	95,996	2.8	95.5	98.3	94.7	94.9	NR	1.8	0.3
Washington^§§§§§^	87,256	97.3	92.5	91.4	91.9	91.3	1.3	3.7	0.4
West Virginia^§§§§,^*****^,§§§§§,†††††^	18,070	85.5	96.5	96.5	96.6	98.0	3.8	0.1	NA
Wisconsin^†††††^	64,275	96.8	≥82.6	≥82.6	≥82.6	≥82.6	8.5	6.3	1.1
Wyoming^††††,§§§§^	7,382	100.0	92.9	92.5	93.8	93.6	2.1	3.9	0.9
**Territories and freely associated states**									
American Samoa^††††^	630	100.0	90.0	94.3	97.0	76.8	NR	0	NA
Federated States of Micronesia^††††^	1,884	100.0	85.4	78.1	82.5	Nreq	NR	NR	NA
Guam^§§§§^	2,236	96.8	91.5	89.8	90.9	Nreq	NR	0.2	NA
Marshall Islands^††††^	1,003	100.0	97.7	93.2	97.3	Nreq	NR	NR	NA
Northern Mariana Islands^††††^	914	100.0	94.4	85.0	90.8	93.5	NR	0	0
Palau	NR	NR	NR	NR	NR	NR	NR	NR	NA
Puerto Rico^§§§§^	27,591	8.0	85.2	92.6	91.2	86.0	NR	1.8	NA
U.S. Virgin Islands	NR	NR	NR	NR	NR	NR	NR	NR	NA

**Abbreviations:** DTaP = diphtheria, tetanus, and acellular pertussis vaccine; DTP = diphtheria and tetanus toxoids and pertussis vaccine; MMR = measles, mumps, and rubella vaccine; polio = poliovirus vaccine; NA = not available; NP = no grace period or provisional policy; NR = not reported to CDC; Nreq = not required; VAR = varicella vaccine.

* Estimates adjusted for nonresponse and weighted for sampling where appropriate.

^†^ Estimates based on a completed vaccination series (i.e., not vaccine specific) use the “≥” symbol. Coverage might include history of disease or laboratory evidence of immunity. In Kentucky, public schools reported numbers of children up to date with specific vaccines, and most private schools reported numbers of children who received all doses of all vaccines required for school entry.

^§^ A grace period is a set number of days during which a student can be enrolled and attend school without proof of complete vaccination or exemption. Provisional enrollment allows a student without complete vaccination or exemption to attend school while completing a catch-up vaccination schedule. In states with one or both of these policies, the estimates represent the number of kindergartners who were within a grace period, were provisionally enrolled, or were in a combination of these categories.

^¶^ Some programs did not report the number of children with exemptions, but instead reported the number of exemptions for each vaccine, which could count some children more than once. Lower bounds of the percentage of children with any exemptions were estimated using the individual vaccines with the highest number of exemptions. Estimates based on vaccine-specific exemptions use the “≥” symbol.

** Exemptions, grace period or provisional enrollment, and vaccine coverage status might not be mutually exclusive. Some children enrolled under a grace period or provisional enrollment might be exempt from one or more vaccinations, and children with exemptions might be fully vaccinated with one or more required vaccines.

^††^ Includes five territories and three freely associated states.

^§§^ The kindergarten population is an approximation provided by each program.

^¶¶^ The number surveyed represents the number surveyed for coverage. Exemption estimates are based on 29,010 kindergartners for Kansas, 58,276 for South Carolina, and 92,265 for Virginia.

*** Most states require 2 doses of MMR; Alaska, New Jersey, and Oregon require 2 doses of measles, 1 dose of mumps, and 1 dose of rubella vaccines. Georgia, New York, New York City, North Carolina, and Virginia require 2 doses of measles and mumps vaccines and 1 dose of rubella vaccine. Iowa requires 2 doses of measles vaccine and 2 doses of rubella vaccine.

^†††^ Pertussis vaccination coverage might include some DTP doses if administered in another country or by a vaccination provider who continued to use DTP after 2000. Most states require 5 doses of DTaP for school entry, or 4 doses if the fourth dose was received on or after the fourth birthday; Maryland and Wisconsin require 4 doses; Nebraska requires 3 doses. The reported coverage estimates represent the percentage of kindergartners with the state-required number of DTaP doses, except for Kentucky, which requires ≥5 but reports ≥4 doses of DTaP.

^§§§^ Most states require 4 doses of polio for school entry, or 3 doses if the fourth dose was received on or after the fourth birthday; Maryland and Nebraska require 3 doses. The reported coverage estimates represent the percentage of kindergartners with the state-required number of polio doses, except for Kentucky, which requires ≥4 but reports ≥3 doses of polio.

^¶¶¶^ Most states require 2 doses of VAR for school entry; Alabama, Arizona, New Jersey, Oklahoma, and Oregon require 1 dose. Reporting of VAR status for kindergartners with a history of varicella disease varied within and among states; some kindergartners were reported as vaccinated against varicella and others as medically exempt.

**** National coverage estimates and medians were calculated using data from 49 states (i.e., do not include Montana) and the District of Columbia. National grace period or provisional enrollment estimates and median were calculated using data from the 27 states that have either a grace period or provisional enrollment policy and reported relevant data to CDC. National exemption estimate and median were calculated from data from 49 states (i.e., did not include Montana) and the District of Columbia. Other jurisdictions excluded were Houston, New York City, American Samoa, Guam, Marshall Islands, Federated States of Micronesia, Northern Mariana Islands, Palau, Puerto Rico, and the U.S. Virgin Islands. Data reported from 3,543,080 kindergartners were assessed for coverage, 3,688,904 for exemptions, and 2,487,284 for grace period or provisional enrollment. Estimates represent rates for populations of coverage (3,837,259), exemptions (3,837,259), and grace period or provisional enrollment (2,607,001).

^††††^ The proportion surveyed is reported as 100% but might be <100% if based on incomplete information about the actual current enrollment.

^§§§§^ Philosophical exemptions were not allowed.

^¶¶¶¶^ Reported public and homeschool school data only.

***** Religious exemptions were not allowed.

^†††††^ Counted some or all vaccine doses received regardless of Advisory Committee on Immunization Practices recommended age and time interval; vaccination coverage rates reported might be higher than those for valid doses.

^§§§§§^ Did not include certain types of schools, such as kindergartens in child care facilities, online schools, correctional facilities, or those located on military bases or tribal lands.

^¶¶¶¶¶^ Vaccination coverage data were collected from a sample of kindergartners; exemption data were collected from a census of kindergartners.

Overall, 2.6% of kindergartners had an exemption (0.2% medical and 2.3% nonmedical^§§§§^) for one or more required vaccines (not limited to MMR, DTaP, polio, and varicella vaccines) in 2021–22 (range = 0.1% [Mississippi, New York, and West Virginia] to 9.8% [Idaho]), compared with 2.2% reported during the 2020–21 school year (Supplementary Table 2, https://stacks.cdc.gov/view/cdc/123204). Among 27 states reporting data on provisional kindergarten enrollment or grace period attendance, 2.4% of children were so enrolled (range = <0.1% [Hawaii] to 8.5% [Wisconsin]).

Nationally, MMR coverage for both the 2020–21 and 2021–22 school years was lower than that reported since 2013–14 (Figure 1). Nationwide, 3.9% of kindergarten students were not fully vaccinated and not exempt. Among the 36 states and the District of Columbia with MMR coverage <95%, all but four could potentially achieve ≥95% MMR coverage if all nonexempt kindergartners who were within a grace period, provisionally enrolled, or otherwise enrolled in school without documentation of vaccination were vaccinated (Figure 2).

**FIGURE 1:**
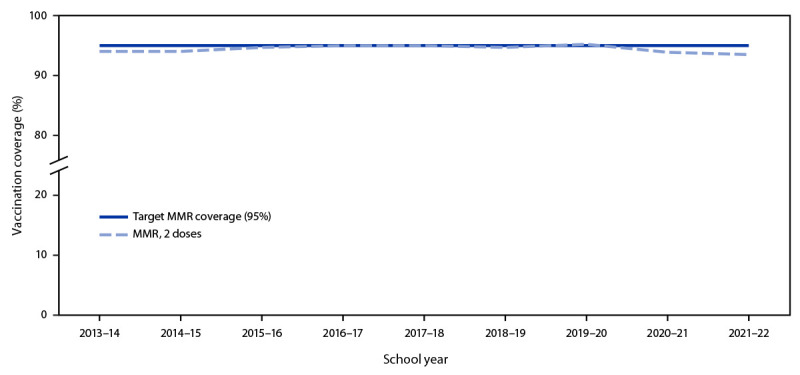
Estimated national coverage with 2 doses of measles, mumps, and rubella vaccine among kindergartners — United States, 2013–14 to 2021–22 school years **Abbreviation:** MMR = measles, mumps, and rubella vaccine.

**FIGURE 2:**
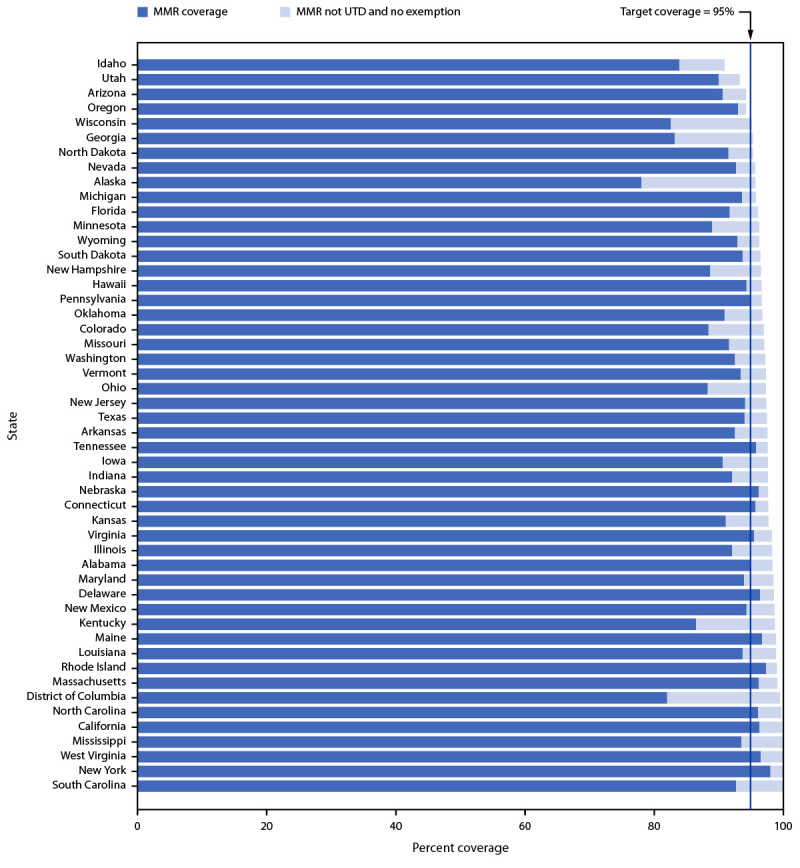
Potentially achievable coverage*^,^^†^ with measles, mumps, and rubella vaccine among kindergartners, by state — United States, 2021–22 school year **Abbreviations**: MMR = measles, mumps, and rubella vaccine; UTD = up to date. * States are ranked from lowest to highest potentially achievable coverage. Potentially achievable coverage is estimated as the sum of the percentage of students with UTD MMR and the percentage of students without UTD MMR and without a documented vaccine exemption. ^†^ The exemptions used to calculate the potential increase in MMR coverage for Alaska, Arizona, Arkansas, Colorado, Delaware, District of Columbia, Idaho, Illinois, Maine, Massachusetts, Michigan, Minnesota, Missouri, Nebraska, Nevada, New York, North Carolina, North Dakota, Ohio, Oklahoma, Oregon, Rhode Island, South Carolina, Texas, Utah, Vermont, Washington, Wisconsin, and Wyoming are the number of children with exemptions specifically for MMR. For all other states, numbers are based on an exemption to any vaccine.

Twenty-three states reported COVID-19–related impacts on data collection including lower response rates from schools, data collection extensions and delays, and incomplete data from schools that did respond; 30 states reported lingering COVID-19–related impacts on vaccination coverage, mostly related to reduced access to vaccination appointments and local or school level extensions of grace period or provisional enrollment policies (CDC, School Vaccination Coverage Report, unpublished data, 2022).

## Discussion

During the 2021–22 school year, coverage with MMR, DTaP, polio, and varicella vaccines among kindergarten children was approximately 93% nationwide for each vaccine, lower than the 94% coverage reported during the 2020–21 school year, and the 95% coverage reported during the 2019–20 school year, when children were vaccinated before the pandemic (*2*,*3*). Coverage with all four vaccines declined in most states. National MMR coverage among kindergarten students remained below the Healthy People 2030 target of 95% (*4*) for the second consecutive year. These findings are consistent with those of continuing declines in routine childhood and adolescent vaccine administration through March 2021 (*5*). MMR coverage of 93.5% translates to nearly 250,000 kindergartners who are potentially not protected against measles; clusters of unvaccinated and undervaccinated children can lead to outbreaks of vaccine-preventable diseases.

The overall percentage of children with an exemption remained low during the 2021–22 school year at 2.6%, although the percentage of children with exemptions increased in 38 states and the District of Columbia. Nationwide, 3.9% of kindergarten students were not fully vaccinated with MMR and not exempt, and this percentage increased in most states compared with 2020–21. Nonexempt undervaccinated students often attend school while in a grace period or are provisionally enrolled; in many states, these policies were expanded either formally or informally during the 2020–21 school year, and this expansion continued to a lesser extent during the 2021–22 school year, even as most schools returned to in-person classes. States continued to report COVID-19–related impacts on vaccination coverage and assessment activities.

The findings in this report are subject to at least five limitations. First, comparisons among states are limited because of variation in states’ requirements such as which vaccines are required, the number of doses required, the date required, and the type of documentation accepted; data collection methods; allowable exemptions; and definitions of grace period and provisional enrollment. Second, representativeness might be negatively affected by data collection methods that assess vaccination status at different times or miss some schools or students, such as those who are homeschooled. Third, vaccination coverage, exemption rates, or both, might be underestimated or overestimated because of inaccurate or absent documentation or missing schools. Fourth, national coverage estimates for the 2021–22 school year include only 49 of 50 states and the District of Columbia and use lower bound estimates for eight states; exemption estimates include 49 states and the District of Columbia and use lower bound estimates for five states, and grace period or provisional enrollment estimates include only 27 states. Finally, states continued to report that the COVID-19 pandemic response created various barriers that limited the amount and quality of student vaccination data collected and reported by local health departments.

Vaccination coverage among kindergarten students remains below prepandemic levels; pockets of undervaccinated children within larger areas of high vaccination coverage can lead to outbreaks (*6*–*8*). Immunization programs can use local-level data, such as that from school assessments or immunization information systems, to identify schools or communities with low vaccination coverage. Rigorously enforced school vaccination requirements, school-based vaccination clinics, reminder and recall systems, and follow-up with undervaccinated students by school nurses are effective strategies to improve vaccination coverage (*9*,*10*). As schools return to in-person learning, high vaccination coverage is critical to continue protecting children and communities from vaccine-preventable diseases.

**Summary**

**What is already known about this topic?**

During the 2020–21 school year, national coverage with state-required vaccines among kindergarten students declined from 95% to approximately 94%.

**What is added by this report?**

During the 2021–22 school year, coverage decreased again to approximately 93% for all state-required vaccines. The exemption rate remained low (2.6%). An additional 3.9% without an exemption were not up to date with measles, mumps and rubella vaccine. Despite widespread return to in-person learning, COVID-19–related disruptions continued to affect vaccination coverage and assessment for the 2021–22 school year, preventing a return to prepandemic coverage.

**What are the implications for public health practice?**

Increasing follow-up with undervaccinated students to reduce the impact of disruptions on vaccination coverage can help protect students from vaccine-preventable diseases.

## Footnotes

* Federally funded immunization programs are located in 50 states and the District of Columbia, five cities, and eight U.S territories and freely associated states. Two cities reported data, which were also included in data submitted by their state, to CDC. State-level data were used to calculate national estimates and medians. Immunization programs in territories reported vaccination coverage and exemptions; however, these data were not included in national calculations.

† Montana did not report school vaccination data.

§ Arkansas, California, Colorado, Florida, Georgia, Hawaii, Idaho, Iowa, Michigan, Mississippi, Nebraska, Nevada, New Hampshire, New Jersey, New Mexico, New York, North Carolina, Ohio, South Carolina, Tennessee, Texas, Utah, Vermont, Washington, West Virginia, Wisconsin, and Wyoming reported data on the number of students within a grace period or provisionally enrolled at the time of assessment.

¶ All states require 2 doses of a measles-containing vaccine. Seven states (Alaska, Georgia, New Jersey, New York, North Carolina, Oregon, and Virginia) require only 1 dose of rubella vaccine. Alaska, New Jersey, and Oregon require only 1 dose of mumps vaccine; mumps vaccine is not required in Iowa.

** Nebraska requires 3 doses of DTaP, Maryland and Wisconsin require 4 doses, and all other states require 5 doses, unless dose 4 was administered on or after the fourth birthday. The reported coverage estimates represent the percentage of kindergartners with the state-required number of DTaP doses, except for Kentucky, which requires 5 doses of DTaP by age 5 years but reported 4-dose coverage for kindergartners.

†† Two states (Maryland and Nebraska) require only 3 doses of polio vaccine; all other states require 4 doses unless the last dose was given on or after the fourth birthday.

§§ Five states require 1 dose of varicella vaccine; 44 states and the District of Columbia require 2 doses.

¶¶ Colorado, Illinois, Minnesota, and Missouri did not report the number of kindergartners with an exemption but instead reported the number of exemptions for each vaccine, which could have counted some children more than once. For these states, the percentage of kindergartners exempt from the vaccine with the highest number of exemptions by exemption type (the lower bound of the potential range of exemptions) was included in the national and median exemption rates.

*** Twelve states reported coverage and exemption data for at least some homeschooled kindergartners, either separately or included with data from public or private schools.

††† Assessment date varied by state and area. Three states assessed schools on the first day of school; 10 states assessed schools by December 31; 18 states and the District of Columbia assessed schools by some other date, ranging from October 15, 2021, to June 23, 2022; and 18 states assessed schools on a rolling basis.

§§§ Immunization programs that used census or voluntary response provided CDC with data aggregated at the state or local (city or territory) level. Estimates based on these data were adjusted for nonresponse using the inverse of the response rate, stratified by school type (public, private, and homeschool, where available). Programs that used complex sample surveys provided CDC with data aggregated at the school or county level for weighted analysis. Weights were calculated to account for sample design and adjusted for nonresponse.

¶¶¶ These totals are the summations of the kindergartners surveyed among programs reporting data for coverage, exemptions, grace periods, and provisional enrollment. Data from cities and territories were not included in these totals.

**** 45 C.F.R. part 46.102(l)(2), 21 C.F.R. part 56; 42 U.S.C. Sect. 241(d); 5 U.S.C. Sect. 552a; 44 U.S.C. Sect. 3501 et seq.

†††† Alabama, Florida, Georgia, Iowa, Mississippi, New Hampshire, New Jersey, and Wisconsin considered kindergartners up to date only if they had received all doses of all vaccines required for school entry. In Kentucky, public schools reported numbers of children up to date with specific vaccines and most private schools reported numbers of children who received all doses of all vaccines required for school entry.

§§§§ Washington was unable to deduplicate data for students with both religious and philosophical exemptions; therefore, the nonmedical exemption type with the highest number of kindergartners (the lower bound of the potential range of nonmedical exemptions) was included in the national and median exemption rates for nonmedical exemptions.
