# *Erratum:* Vol. 72, No. 2

**DOI:** 10.15585/mmwr.mm7210a5

**Published:** 2023-03-10

In the report “Vaccination Coverage with Selected Vaccines and Exemption Rates Among Children in Kindergarten — United States, 2021–22 School Year,” multiple errors occurred. On page 26, in the eighth line of the first paragraph, the sentences should have read, “Nationwide, vaccination coverage with 2 doses of measles, mumps and rubella vaccine (MMR) was **93.0**%^¶^; with the state-required number of diphtheria, tetanus, and acellular pertussis vaccine (DTaP) doses was **92.7**%**; with poliovirus vaccine (polio) was **93.1%**^††^; and with the state-required number of varicella vaccine doses was 92.8%.^§§^ Compared with the 2020–21 school year, vaccination coverage decreased **0.8–0.9** percentage points for all vaccines. Although 2.6% of kindergartners had an exemption for at least one vaccine,^¶¶^ an additional **4.4**% who did not have an exemption were not up to date with MMR.”

On page 27, in the fifth line of the first paragraph, the sentences should have read, “During the 2021–22 school year, immunization programs reported **3,835,130** children enrolled in kindergarten in 49 states and the District of Columbia.^¶¶¶^ Reported estimates are based on **3,536,546** (92.2%) children who were surveyed for vaccination coverage, **3,686,775 (96.1%)** surveyed for exemptions, and **2,527,578 (65.9%)** surveyed for grace period and provisional enrollment status.” Also on page 27, in the first line of the second column, the sentences should have read, “Nationally, 2-dose MMR coverage was **93.0**% (range = 78.0% [Alaska] to **≥98.6% [Mississippi]**), with coverage of ≥95% reported by **14** states and <90% by nine states and the District of Columbia (Table). DTaP coverage was **92.7**% (range = 78.0% [Alaska] to **≥98.6% [Mississippi]**); coverage of ≥95% was reported by **15** states and of <90% by 12 states and the District of Columbia. Polio vaccination coverage was **93.1**% (range = 77.1% [Alaska] to **≥98.6% [Mississippi]**), with coverage of ≥95% reported by **14** states and <90% by 10 states and the District of Columbia. Varicella vaccination coverage nationally was 92.8% (range = 76.1% [Alaska] to **≥98.6% [Mississippi]**), with **13** states reporting coverage ≥95% and nine states and the District of Columbia reporting <90% coverage.” Also on page 27, in the third line of the third paragraph in the second column, the sentences should have read, “Nationwide, **4.4**% of kindergarten students were not fully vaccinated and not exempt. Among the **35** states and the District of Columbia with MMR coverage <95%, all but four could potentially achieve ≥95% MMR coverage if all nonexempt kindergartners who were within a grace period, provisionally enrolled, or otherwise enrolled in school without documentation of vaccination were vaccinated (Figure 2).” 

On page 28, the Table contained multiple errors. In the first row, labeled “National estimate,” the value under the column heading “Kindergarten population” should have been **3,835,130**, the value under the heading “2 Doses MMR” should have been **93.0**, the value under the heading “5 Doses DTaP” should have been **92.7**, and the value under the heading “4 Doses polio” should have been **93.1**. In the 28th row, labeled “Mississippi,” the value under the column heading “Kindergarten population” should have been **36,524**; the value under the heading “2 Doses MMR” should have been **≥98.6**; the value under the heading “5 Doses DTaP” should have been **≥98.6**; the value under the heading “4 Doses polio” should have been **≥98.6**; the value under the heading “2 Doses VAR” should have been **≥98.6**; and the value under the heading “Grace period or provisional enrollment, %” should have been **1.0**. 

On page 29, the last two sentences in the 13th footnote should have read, “****Data reported from **3,536,546** kindergartners were assessed for coverage, **3,686,775** for exemptions, and **2,527,578** for grace period or provisional enrollment. Estimates represent rates for populations of coverage (**3,835,130**), exemptions (**3,835,130**), and grace period or provisional enrollment (**2,604,872**).” 

On page 30, in the 13th line of the first paragraph, the sentences should have read, “MMR coverage of **93.0**% translates to **approximately** 250,000 kindergartners who are potentially not protected against measles; clusters of unvaccinated and undervaccinated children can lead to outbreaks of vaccine-preventable diseases.” Also on page 30, in the fourth line of the second paragraph, the sentence should have read, “Nationwide, **4.4**% of kindergarten students were not fully vaccinated with MMR and not exempt, and this percentage increased in most states compared with 2020–21.”

On page 30, in Figure 1, the line for “MMR, 2 doses” for 2021–2022, should have indicated a value of **93.0%**.

On page 31, in Figure 2, the bar for “MMR coverage” among kindergartners for Mississippi should have indicated a value of **98.6**%. The bar for “MMR not up to date and no exemption” for kindergartners in Delaware should have indicated a value of **2.4**%, and for South Carolina, should have indicated a value of **4.6%**.

On page 32, in the Summary box, the second line in the second paragraph should have read, “An additional **4.4**% without an exemption were not up to date with measles, mumps and rubella vaccine.”

Supplementary materials have also been corrected.

